# A Sneer at Doctors

**Published:** 1920-06-05

**Authors:** 


THE PUBLIC WELL-BEING—(*continued*).

### A Sneer at Doctors.

At the annual conference of the Manchester Unity of Oddfellows, the Grand Master made some ungenerous remarks upon doctors, and criticised them for having "as their principal object, not what could be done to relieve suffering, but how much could be got out of the transaction." Allowance, perhaps, should be made for the ceaseless dislike of the Insurance Act by friendly societies, which has some reflection on the attitude towards doctors. The form of the Grand Master's remarks, however, was unjust. It is a gratuitous assumption that the doctor's first aim is not to relieve suffering, and it is narrow-minded to cavil even if a doctor, like any other ordinary human being, takes what care he can, also, to get as well paid as possible.

There is probably no other professional man who gives so much gratuitous service, has to wait so long for his money, and has so many bad debts as the average medical practitioner. Nobody ever hesitates to call upon him on a football ground, in a concert hall, in the streets, on the railways, or in any other conceivable place, if a doctor's skill is required, and he responds without the slightest guarantee that he will receive any reward for his professional attendance. Few people realise the enormous amount of time given freely to institutional work, and a profession which has these traditions is entitled to protection from cheap sneers. The art of healing is a noble one, but it is not an other-worldly one. It is a means of livelihood like any other calling; and it is a new ethic to suppose that the higher the type of work the less should be the reward. A judge is pretty well paid for dispensing justice, and no one suggests that he is any the worse a judge for that, either morally or professionally; he doesn't, in any case, try legal suits for nothing. Yet the doctor is always doing something for nothing. One of the papers, writing on this subject a short time ago, pointed out that a sculptor never finds anyone lying by the road on the point of perishing unless he can at once be given a small bas-relief for which he is unlikely to pay.

Every doctor is preyed upon to some extent, and there are a good many distinguished men amongst them who, in the ordinary course of their work, do not allow a patient's inability to pay the full fee which can always be commanded to interfere with professional attendance, or the performance of an operation. Every man tries to get the best reward for his labour, and every honourable man tries to give his best in return. There are plenty of people —unnecessary to name—who make no bones about getting all they can, by force, for as little as possible; and it would be hard to find anything that they do for nothing. It is particularly unfair to exercise spleen on the medical profession, whose record will bear comparison with that of any other body of men, and will put to shame the record of many of them.

